# Proporção de Largura de Distribuição de Hemoglobina/Glóbulos Vermelhos (HRR): Um Novo e Promissor Parâmetro de Glóbulos Vermelhos no Fechamento Ductal

**DOI:** 10.36660/abc.20220339

**Published:** 2023-02-13

**Authors:** Duran Yildiz, Ufuk Cakir, Ali Ulaş Tugcu, Burak Ceran, Cuneyt Tayman

**Affiliations:** 1 Divisão de Neonatologia Departamento de Pediatria Universidade de Ciências da Saúde Ankara Turkey Ankara Bilkent City Hospital – Divisão de Neonatologia – Departamento de Pediatria – Universidade de Ciências da Saúde, Ankara – Turkey

**Keywords:** Cardiopatias Congênitas, Permeabilidade do Canal Arterial, Recém-nascido Prematuro, Hemoglobina

## Abstract

**Fundamento:**

Ainda não está clara a eficácia dos parâmetros hematológicos no fechamento da persistência do canal arterial (PCA).

**Objetivos:**

O objetivo principal do nosso estudo é investigar o efeito da proporção (HRR) de largura de distribuição de hemoglobina (HB) para glóbulos vermelhos (RDW) no fechamento do PCA.

**Métodos:**

Bebês prematuros com muito baixo peso ao nascer (MBPN: <1.500 g) e <32 semanas gestacionais foram incluídos no estudo, e todos os dados foram registrados retrospectivamente. Características demográficas, resultados clínicos, parâmetros de hemácias e HRR e suas proporções foram comparados entre grupos de PCA hemodinamicamente significativa (hsPDA) e não-hsPDA. Todos os resultados foram analisados estatisticamente, e p<0,05 foi considerado estatisticamente significativo.

**Resultados:**

Um total de 677 bebês prematuros, 269 no grupo hsPDA e 408 no grupo não-hsPDA, foram incluídos no estudo. Hemoglobina (HB), hematócrito (HCT), volume celular médio (VCM), glóbulos vermelhos (RBC), largura de distribuição dos glóbulos vermelhos (RDW), volume plaquetário médio (VPM), relação VCM/RBC, relação HB/RBC, RDW A razão /RBC e a razão RDW/VPM foram semelhantes entre os grupos hsPDA e não hsPDA, (p>0,05). HRR foi significativamente menor no grupo hsPDA [mediana (Quartil 1 (Q1) - Q3) (Q1 - Q3): 0,93 (0,8-1,0)] em comparação com não-hsPDA [mediana (Q1 - Q3): 1,07 ( 1,0-1,2)] (p<0,001). A AUC para o valor diagnóstico de HRR em hsPDA foi de 0,816 e o valor de corte foi ≤0,98 (p<0,001, 95% [IC]: 0,785-0,845, sensibilidade: 90%, especificidade: 92%).

**Conclusões:**

O valor de HRR foi considerado um parâmetro eficaz e poderoso no diagnóstico de hsPDA.

## Introdução

A persistência do canal arterial (PCA) é a doença cardíaca mais comum em prematuros. Há uma taxa crescente de morbidade e mortalidade relacionada à PCA em prematuros. O shunt da esquerda para a direita devido à PCA causa diminuição do fluxo sanguíneo sistêmico e aumento do fluxo sanguíneo pulmonar. Como resultado, há um risco aumentado de morbidades graves, como edema pulmonar e sangramento, aumento da necessidade de suporte respiratório, hemorragia intraventricular (HIV), leucomalácia periventricular, enterocolite necrosante (ECN), retinopatia da prematuridade (ROP), displasia broncopulmonar (DBP) e resultados de neurodesenvolvimento ruins.

A falha no fechamento do canal arterial (CA) dentro de 72 horas é definida como PCA. A frequência de PCA é inversamente proporcional à semana gestacional (SG) e ao peso ao nascer (PN). A incidência de PCA é de 60-70% em prematuros com menos de 28 SG e 20% em prematuros <32 semanas de gestação. A PCA é observada em uma taxa de 40-55% em bebês com peso corporal <1.000 g e 30% em bebês com peso corporal <1.500 g.^3^

O fechamento funcional do DA ocorre logo após o nascimento devido ao aumento da saturação de oxigênio e diminuição dos níveis de prostaglandina. Dano endotelial, ruptura subintimal, interação plaqueta-endotelial, proliferação neointimal e formação de estrutura fibrosa vascular são eficazes no fechamento anatômico. Embora os efeitos de SG, PN e os mecanismos acima no fechamento

ductal sejam conhecidos, outros mecanismos que afetam o fechamento ductal não são totalmente compreendidos.^1^

Alguns marcadores e parâmetros bioquímicos podem afetar o fechamento ductal.^4 - 6^ Além disso, os parâmetros hematológicos também podem afetar o fechamento ductal. Muitos estudos avaliam a relação entre contagem de plaquetas, índices e funções e PCA.^1 , 7 , 8^ No entanto, existem poucos estudos sobre o efeito dos eritrócitos e parâmetros relacionados no fechamento ductal.^9 , 10^ Atualmente, o efeito dos parâmetros hematológicos no PCA não foi totalmente elucidado. Um pequeno número de estudos em adultos concluiu que a proporção (HRR) da largura de distribuição (RDW) da hemoglobina (HB) para os glóbulos vermelhos pode ser um marcador prognóstico em alguns tipos de câncer.^11 - 13^ Estudos recentes determinaram que o baixo valor de HRR em adultos com insuficiência cardíaca e doença cardíaca coronária é um fator de risco independente para mortalidade e resultados clínicos adversos.^14 - 16^ No entanto, não foi avaliado se a HRR pode ser um parâmetro preditor na PCA, que é o problema cardiovascular mais comum em recém-nascidos.

O significado clínico de HRR na PCA não foi previamente avaliado. De acordo com a hipótese de nosso estudo, a HRR derivada de parâmetros hematológicos que podem afetar o fechamento ductal pode ser um novo parâmetro promissor. O objetivo principal deste estudo é avaliar a relação entre HRR e PCA. O objetivo secundário do nosso estudo é avaliar a relação entre PCA e outros parâmetros de hemácias no hemograma completo.

## Métodos

### Plano de estudo e seleção de pacientes

Bebês prematuros com PN <1.500 g e SG <32 semanas foram elegíveis para o estudo. O estudo foi retrospectivo e os dados foram obtidos dos prontuários do hospital entre setembro de 2020 e novembro de 2021. Pacientes com grandes anomalias congênitas, cardiopatia congênita, asfixia perinatal, peso ao nascer ≥1.500 g e pacientes que morreram nos primeiros três dias após nascimento foram excluídos do estudo. As características demográficas e clínicas dos pacientes e os parâmetros dos glóbulos vermelhos no hemograma completo foram registrados. Os pacientes foram divididos em PCA hemodinamicamente significativa (hsPDA) e não hsPDA. A aprovação ética foi obtida do comitê de ética do hospital local antes de iniciar o estudo.

### Características demográficas e clínicas

Semana gestacional, PN, sexo, administração pré-natal de esteroides, escores de Apgar (aos 1 e 5 minutos), pequeno para a idade gestacional (PIG), síndrome do desconforto respiratório (SDR), HIV (grau ≥3), ECN (grau >2), DBP moderada/grave, ROP (requer terapia), sepse neonatal precoce (SNNP), sepse neonatal tardia (SNNT), ventilação mecânica (VM), ventilação não invasiva (VNI) e duração do oxigênio, alcançando o tempo total de alimentação enteral (dia), a duração da internação na unidade de terapia intensiva neonatal (UTIN) e as taxas de mortalidade foram registradas para todos os bebês.

Os pacientes que necessitam de tratamento com surfactante foram definidos como SDR.^17^ O diagnóstico de HIV grave (grau ≥3) foi demonstrado por ultrassonografia craniana.^18^ A classificação ECN (grau ≥2) foi feita com base em achados clínicos e laboratoriais.^19^ A DBP foi definida como pacientes com necessidade de oxigênio <30% (moderada) ou uso de oxigênio ≥30% ou pressão positiva (grave) na 36ª semana de idade pós-menstrual.^20^ Foram registradas as pacientes diagnosticadas com ROP de acordo com exame de retina realizado por um oftalmologista e posteriormente tratadas.^21^ Bebês com peso ao nascer abaixo do percentil 10 para SG foram definidos como PIG.^22^ Sepse pós-natal foi definida como SNNP se fosse ≤72 horas e SNNT se fosse >72 horas.^23^

### Diagnóstico de persistência do canal arterial hemodinamicamente significativa

Seguindo o protocolo de nossa unidade, todos os pacientes do estudo recebem ecocardiografia Doppler (ECO) a cada 2 dias durante a primeira semana, a partir das primeiras 72 horas. Os pacientes foram diagnosticados com hsPDA de acordo com os critérios clínicos e ECO na Tabela 1 .^2^ Se o diâmetro interno da PCA fosse <1,5 mm e/ou a relação átrio esquerdo/raiz da aorta fosse <1,5 por ECO, ou se a PCA não fosse detectada e se clinicamente e ecocardiograficamente fosse diferentes do hsPDA, esses pacientes foram incluídos no grupo não-hsPDA. Os pacientes do grupo hsPDA receberam terapia medicamentosa (anti-inflamatório não esteroide) pelo menos uma vez.^24^ Aqueles que não responderam a dois cursos de tratamento médico foram tratados cirurgicamente. A ingestão de líquidos foi iniciada em 70-80 mL/kg por dia em todos os lactentes e aumentada até um máximo de 150-160 mL/kg por dia em incrementos de 10-20 mL/kg por dia para todos os pacientes elegíveis.

**Tabela 1:** – Persistência do canal arterial hemodinamicamente significativa

Características clínicas	Murmúrio
	Precórdio hiperdinâmico
	Pulsos pré-ductais delimitadores
	Piora do estado respiratório
	Pressão de pulso largo
	Hipotensão
	Acidose metabólica
**Recursos ecocardiográficos**	Aumento da relação entre o átrio esquerdo e a raiz da aorta
	Cardiomegalia
	Shunt da esquerda para a direita
	Grande ducto aberto (>1,5 mm)
	Reversão do fluxo nas artérias principais pós-ductais

### Análise de hemograma completo

De acordo com o protocolo da unidade, amostras de sangue da veia umbilical foram coletadas em tubos de ácido etilenodiamino tetra-acético (EDTA) logo após o parto para um hemograma completo. Os valores de HB (g/dl), hematócrito (HCT, %), volume celular médio (VCM, fL), glóbulos vermelhos (RBC, 106/µL), RDW (%) e volume plaquetário médio (VPM) foram analisados com hemocitômetro automático Cell-Dyn 3700 (Abbott, Abbott Park, IL, EUA). O valor da HRR foi obtido dividindo HB por RDW. O valor da relação VCM/RBC foi obtido dividindo o VCM por RBC. A relação HB/RBC foi obtida dividindo HB por RBC. A razão RDW/RBC foi obtida dividindo RDW por RBC. A relação RDW/VPM foi obtida dividindo RDW por VPM.

### Análise estatística

Após a transferência dos dados do paciente para o ambiente computacional, a análise estatística foi realizada com o programa Statistical Package for the Social Sciences (SPSS), versão 20.0 (SPSS Inc, Chicago, IL, EUA). Métodos visuais (gráficos de probabilidade e histograma) e analíticos (Teste de Kolmogorov-Smirnov) foram usados para avaliar as variáveis quanto à sua conformidade com a distribuição normal. O teste exato de Fisher ou o teste qui-quadrado de Pearson foi usado para avaliar as variáveis categóricas. Um teste t de Student não pareado ou teste U de Mann-Whitney foi aplicado para variáveis contínuas. As variáveis contínuas com distribuição normal foram apresentadas como média ± desvio padrão (DP), as variáveis com distribuição não normal foram apresentadas como mediana (Quartil 1 (Q1) - Quartil 3 (Q3)) (Q1 - Q3) e as variáveis categóricas foram apresentadas como frequência. A análise das curvas ROC (Receiver Operating Characteristics) foi realizada. Após a análise ROC, a área sob a curva (AUC) e o intervalo de confiança (IC) de 95% da AUC foram calculados. O limite do valor de HRR para fechamento ductal foi definido. A sensibilidade e a especificidade foram determinadas para o limiar do valor de HRR. Se o valor de p obtido fosse inferior a 0,05, era considerado estatisticamente significativo. O tamanho da amostra foi introduzido para o desfecho primário como fechamento ductal; 124 pacientes em cada grupo teriam 80% de poder para detectar uma diferença de 25% entre os grupos (de 60 a 85%) na porcentagem de fechamentos permanentes, usando um teste χ^2^ bilateral corrigido para continuidade em um nível de significância de 0,05.^8^

## Resultados

**Figura 1:**
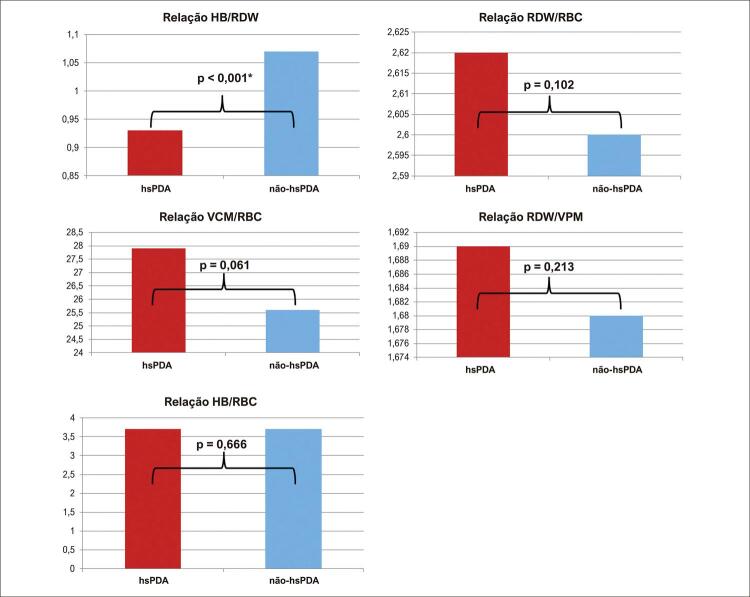
– Relações hematológicas em grupos com e sem persistência do canal arterial hemodinamicamente significativa. HB: hemoglobina; hsPDA: persistência do canal arterial hemodinamicamente significativa; VCM: volume celular médio; VPM: volume plaquetário médio; RBC: glóbulos vermelhos; RDW: largura de distribuição de hemoglobina/glóbulos vermelhos; *Os valores p estatísticos são destacados

**Figura 2:**
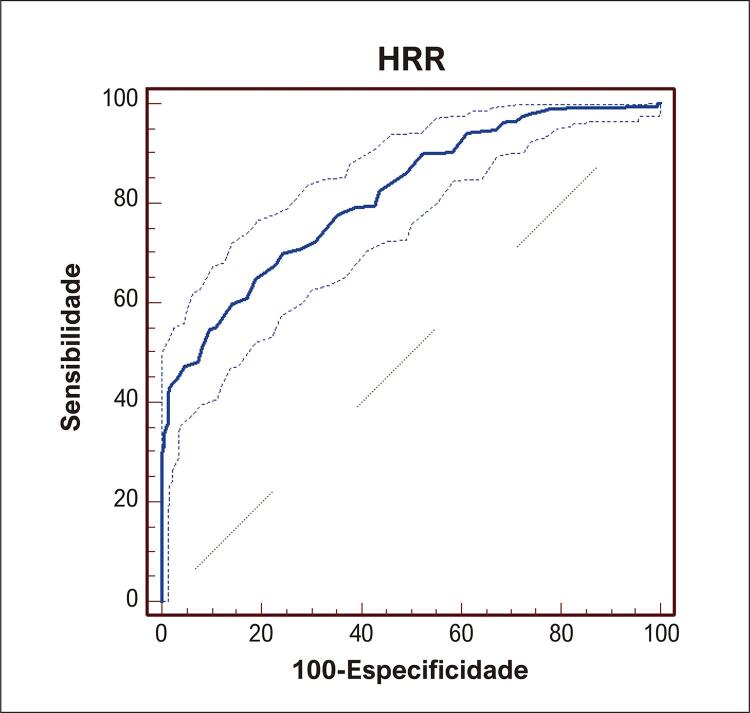
– Curvas características de operação do receptor para HRR em hsPDA. hsPDA: persistência do canal arterial hemodinamicamente significativa; HRR: proporção de largura de distribuição de hemoglobina/glóbulos vermelhos.

Um total de 677 bebês MBPN que preencheram os critérios de inclusão foram alocados para o grupo de estudo. 269 pacientes foram incluídos no grupo hsPDA e 408 no grupo não hsPDA. No grupo hsPDA, o suporte respiratório (VM, VNI e suporte de oxigênio), a obtenção de um dia completo de alimentação enteral e a permanência na UTIN foram mais longos. SDR, DBP, ROP, HIV e frequência de mortalidade foram significativamente maiores (p<0,05). Não houve diferença entre os dois grupos em relação à administração de esteroides pré-natal (p>0,05). Outras características demográficas e clínicas foram semelhantes entre os grupos (p>0,05) ( Tabela 2 ). Dentre os parâmetros hematológicos, apenas a HRR foi significativamente menor no grupo hsPDA do que no grupo não hsPDA (p<0,001). Os resultados dos demais parâmetros hematológicos foram semelhantes entre os grupos (p>0,05) ( Tabela 3 , Figura 1 ). O valor AUC para o valor diagnóstico de HRR em hsPDA foi de 0,816 e o valor de corte foi ≤0,98 (p<0,001, 95% [IC]: 0,785-0,845, sensibilidade: 90%, especificidade: 92%) ( Figura 2 ). Além disso, os resultados de nosso artigo foram resumidos na figura central .

**Tabela 2:** – Características demográficas e clínicas dos grupos hsPDA e não-hs PCA

Variáveis	hsPDA (n: 269, 39,7%)	Não-hsPDA (n: 408, 60,3%)	p
Idade gestacional, semanas^a^	28,2± 2,0	28,5 ± 2,1	0,089
Peso ao nascer, g^a^	1041 ± 220	1080 ± 248	0,103
Masculino^b^	129 (47,9)	220 (53,9)	0,388
Esteroide pré-natal ^b^	183 (69)	290 (71)	0,929
Escore Apgar 1 min ^c^	5 (4-6)	5 (5-6)	0,112
Escore Apgar 5 min ^c^	7 (6-8)	7 (7-8)	0,208
Duração do oxigênio, dias^c^	35 (12-48)	12 (5-29)	<0,001*
Duração VNI, dias^c^	10 (4-17)	4 (1-6)	<0,001*
Duração VM, dias^c^	2 (0-17)	0 (0-2)	<0,001*
SNNP^b^	6 (2,2)	9 (2,2)	0,671
SNNT^b^	75 (27,8)	81 (19,8)	0,086
SDR^b^	217 (80,6)	199 (48,7)	<0,001*
DBP^b^	79 (29,3)	36 (8,8)	<0,001*
ROP^b^	36 (13,3)	19 (4,6)	<0,001*
VIH (grado≥3)^b^	37 (13,7)	19 (4,6)	<0,001*
ECN (grado≥2)^b^	7 (2,6)	7 (1,7)	0,114
Alimentação enteral completa, dia^c^	15 (6-21)	13 (11-17)	0,001*
Permanência na UTIN, dias^c^	67 (45-80)	51 (36-66)	0,001*
Mortalidade^b^	45 (16,7)	53 (12,9)	0,007*

*Valores de p estatisticamente significativos são destacados. ^*a*^ média ± desvio padrão, ^*b*^ n (%), ^*c*^ mediana (Quartil 1 (Q1)- Quartil 3 (Q3)) ( Q1 - Q3). DBP: displasia broncopulmonar; SNNP: sepse neonatal precoce; HIV: hemorragia intraventricular; SNNT: sepse de início tardio; VM: ventilação mecânica; ECN: enterocolite necrosante; UTIN: unidade de terapia intensiva neonatal; VNI: ventilação não invasiva; hsPDA: persistência do canal arterial hemodinamicamente significativo; SDR: síndrome do desconforto respiratório; ROP: retinopatia da prematuridade.

**Tabela 3:** – Parâmetros hematológicos dos grupos hsPDA e não-hs PCA

Variáveis	hsPDA (n: 269, 39,7%)	Não-hsPDA (n: 408, 60,3%)	p
HB (g/dL) ^a^	15,7 (14,4-16,9)	16,7 (15,5-18,1)	0,352
HCT (%) ^a^	48 (44,2-51,0)	51,4 (47,9-55,2)	0,736
VCM (fL) ^a^	114 (109-119)	115 (110-120)	0,146
RBC (106/µL) ^a^	4,17 (3,7-4,4)	4,4 (4,1-4,8)	0,711
VPM (fL) ^a^	7,5 (7,1-8,3)	7,6 (7,4-8,4)	0,321
RDW (%) ^a^	15,9 (15,2-16,5)	15,9 (15,2-16,6)	0,367
Relação HB/RDW ^a^	0,93 (0,8-1,0)	1,07 (1,0-1,2)	<0,001*
Relação VCM/RBC ^a^	27,9 (24,9-30,7)	25,6 (23,0-28,6)	0,061
Relação HB/RBC ^a^	3,7 (3,5-3,9)	3,7 (3,5-3,9)	0,666
Relação RDW/RBC ^a^	2,62 (3,5-4,2)	2,60 (3,3-3,9)	0,102
Relação RDW/VPM ^a^	1,69 (1,8-2,2)	1,68 (1,8-2,1)	0,213

*Valores de p estatisticamente significativos são destacados. ^*a*^ mediana (Quartil 1 (Q1) - Quartil 3 (Q3)) (Q1 - Q3). HB: hemoglobina; HCT: hematócrito; hsPDA: persistência do canal arterial hemodinamicamente significativo; VCM: volume celular médio; VPM: volume plaquetário médio; RBC: glóbulos vermelhos; RDW: largura de distribuição de glóbulos vermelhos.

**Figura Central:**
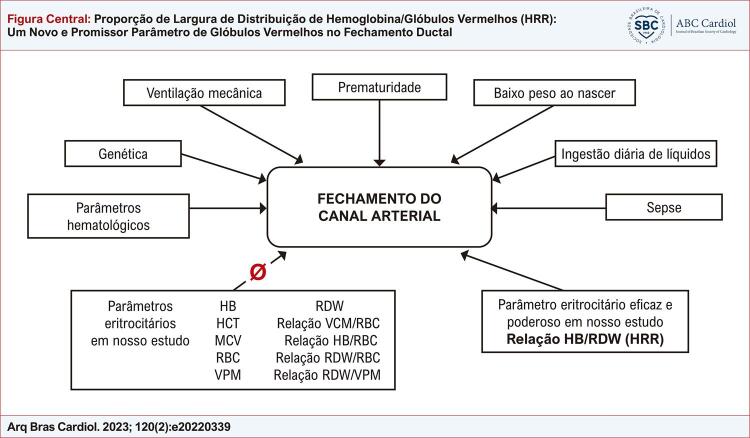
: Proporção de Largura de Distribuição de Hemoglobina/Glóbulos Vermelhos (HRR): Um Novo e Promissor Parâmetro de Glóbulos Vermelhos no Fechamento Ductal

Resumo dos resultados do nosso estudo.

## Discussão

O objetivo principal do nosso estudo foi avaliar a relação da HRR com hsPDA. Este estudo descobriu que o valor de HRR no hemograma completo foi o parâmetro mais valioso para determinar hsPDA, com alta AUC, especificidade e sensibilidade entre os parâmetros de hemácias. Se o valor de HRR for ≤9,8, foi considerado altamente significativo para o risco de hsPDA. No entanto, nenhuma relação foi encontrada entre hsPDA e outros parâmetros de hemácias, que era nosso objetivo secundário. Além disso, a duração do suporte respiratório, o dia completo de alimentação enteral e a permanência na UTI foram maiores em lactentes com hsPDA. Os fatores de risco mais importantes para morbidade e mortalidade prematura foram SG e PN, pois SG e PN foram semelhantes em ambos os grupos. O aumento da taxa de morbidade prematura (SDR, DBP, ROP, HIV) e mortalidade no grupo hsPDA parecia ser devido ao efeito do hsPDA. Portanto, nossos resultados apoiaram o conhecimento de que hsPDA pode aumentar a morbidade e mortalidade prematura em bebês prematuros.^2 , 3^

Se os valores de HB e HCT diminuírem, a hipóxia tecidual aumenta. Sugere-se que o fechamento ductal diminuirá com o aprofundamento da hipóxia. No entanto, o efeito dos valores de HB e HCT no hsPDA não é totalmente conhecido.^2 , 3^ Joye et al.,^10^ relataram um aumento na frequência de hsPDA na presença de anemia grave que requer transfusão pós-parto. No mesmo estudo, interpretou-se que um nível de HB mais alto e estável nos primeiros dez dias de vida pode afetar positivamente o fechamento esperado do CA. Além disso, no estudo mencionado, o nível de HB foi >13,5 g/dL no grupo não hsPDA. Portanto, os autores sugeriram que o fechamento do CA pode ser sustentado por um maior nível de HB ou manutenção de um determinado nível de HB durante o período de transição. Nosso estudo não encontrou relação entre os valores de HB e HCT e hsPDA.

O efeito do VCM no fechamento ductal é desconhecido. Determinamos que o VCM não estava associado ao hsPDA. Assim, concluiu-se que o volume eritrocitário não afetou o fechamento ductal. Além disso, não encontramos nenhuma correlação entre o valor de RBC e hsPDA. A relação entre RBC e PCA não foi avaliada antes. No entanto, Bin-Nun et al.,^25^ descobriram que a contagem absoluta de hemácias nucleadas era significativamente maior no grupo hsPDA do que no grupo não hsPDA. Especula-se que esse resultado se deva ao alto número absoluto de hemácias nucleadas e permeabilidade ductal após o parto devido à exposição à hipóxia fetal.^25^ Outro parâmetro dos glóbulos vermelhos, RDW, reflete a heterogeneidade no volume dos eritrócitos. Em seu estudo envolvendo 41 bebês prematuros, Garofoli et al.,^26^ relataram que pacientes com hsPDA tinham um valor de RDW mais alto, e houve uma relação inversa entre os valores de RDW e SG.^26^ O aumento de RDW em pacientes com hsPDA pode ser decorrente da maior frequência de SNNT no grupo com hsPDA. Acredita-se que o aumento do RDW ocorra devido a citocinas inflamatórias que inibem a maturação eritrocitária e aceleram a transição de reticulócitos mais jovens e maiores para a circulação periférica.^27^ Estudos anteriores, incluindo maior número de pacientes, declararam não haver relação entre RDW e hsPDA. Nesses estudos, foi declarado que a frequência de sepse foi semelhante entre os grupos em paralelo aos nossos resultados.^3 , 17^ Portanto, RDW parece ser afetada principalmente pela inflamação relacionada à sepse em vez de hsPDA.^27^ Acredita-se que o aumento da RDW ocorra devido a citocinas inflamatórias que inibem a maturação eritrocitária e aceleram a transição de reticulócitos mais jovens e maiores para a circulação periférica.^27^ Estudos anteriores, incluindo maior número de pacientes, declararam não haver relação entre RDW e hsPDA.

A possível relação entre a relação VCM/RBC, HB/RBC, RDW/RBC e RDW/VPM com qualquer doença do recém-nascido não foi investigada. Verificou-se que VCM/RBC pode ser usada efetivamente para diferenciar anemia por deficiência de ferro e talassemia na infância.^28^ Também foi demonstrado que a relação RDW/RBC pode ser usada como um parâmetro eficaz para diferenciar beta-talassemia e deficiência de ferro.^29^ A relação RDW/RBC também foi relatada como um teste de triagem de α-talassemia.^30^ Além disso, foi declarado que tanto a relação RDW/RBC quanto RDW/VPM podem ser usadas como indicadores importantes no diagnóstico de apendicite aguda complicada em infância.^31^ A relação HB/RBC só foi estudada na toxicidade hepática. Foi demonstrado que a gravidade da toxicidade hepática e o valor da razão HB/RBC são diretamente proporcionais, sendo um parâmetro eficaz na demonstração do processo hemostático.^32^

Em nosso estudo, a relação entre a relação VCM/RBC, HB/RBC, RDW/RBC e RDW/VPM com hsPDA não pôde ser demonstrada. Os parâmetros usados em todas essas proporções são calculados sobre o volume ou largura de distribuição do eritrócito. O volume ou largura de distribuição dos eritrócitos muda em doenças onde a inflamação está em primeiro plano, incluindo sepse. Portanto, essas proporções provavelmente são um indicador de inflamação. Em nossos resultados, a incidência de sepse foi semelhante nos grupos com e sem hsPDA. Esse resultado sugere que a frequência de inflamação foi semelhante entre os grupos. Nossos resultados apoiaram a informação de que a relação VCM/RBC, relação HB/RBC, relação RDW/RBC e relação RDW/VPM foram particularmente afetadas em doenças causadas por inflamação.^27^ Portanto, as pesquisas continuam para outro parâmetro hematológico relacionado e preditivo que não é afetado pela inflamação para PCA, que é afetado principalmente por SG e PN e tem uma fisiopatologia mista. O fato de o parâmetro requerido estar associado à oxigenação tecidual, que tem efeito significativo no fechamento do CA, pode aumentar o valor de uso deste parâmetro. Portanto, o valor de HB tem uma relação paralela com a oxigenação tecidual; pode ser um parâmetro seguro na avaliação da relação entre HB e CA. Como a relação entre HB e PCA não é conhecida exatamente, o efeito dos parâmetros, incluindo HB, no processo de fechamento do duto deve ser avaliado. Em adultos, o significado prognóstico da HRR em pacientes com câncer tem sido objeto de estudos limitados. Foi relatado que está associado a um mau prognóstico de câncer abaixo de certos valores limiares para certos tipos de câncer. Por exemplo, valores de HRR de <0,989 em câncer de células escamosas de esôfago, <1,037 em câncer de cabeça e pescoço, <0,94 em câncer de bexiga músculo-invasivo, <0,948 em câncer de pulmão de células não pequenas e <1,01 em adenocarcinoma de pulmão são indicadores independentes para fatores de mau prognóstico dos pacientes.^11 - 13 , 33 , 34^ No estudo de Rahamim et al.,^14^ no qual 6.888 pacientes adultos com insuficiência cardíaca foram avaliados, foi relatado que a HRR foi um parâmetro mais forte na predição de mortalidade do que a HB ou apenas RDW.^14^ A baixa HRR pode ser um preditor independente e forte de mortalidade e resultados clínicos adversos na doença coronariana. Além disso, HRR <9,76 foi o valor limite para o estado de fragilidade em doenças coronarianas.^15 , 16^ De acordo com nossos resultados, o valor de HRR < 0,98 obtido do cordão umbilical foi o parâmetro mais significativo para predizer hsPDA. Por outro lado, embora HB ou RDW sozinhos não tenham sido significativos na predição de hsPDA, descobriu-se que o valor de HRR obtido da razão desses dois parâmetros pode ser o marcador derivado de eritrócitos mais significativo e mais forte.

Em estudos anteriores em pacientes com câncer, foi relatado que a HRR é um indicador do estado imunológico, nutricional e inflamatório.^11^ Quando HB e RDW foram usados como fatores prognósticos em estudos sobre tipos de câncer, nenhuma diferença significativa foi encontrada na sobrevida global. Em vez de avaliar apenas HB e RDW, como em nossos resultados, seu valor clínico torna-se significativo quando usado como HRR, que é uma razão entre si.^34^ Resultados semelhantes foram obtidos em estudos avaliando a relação entre insuficiência cardíaca e doenças coronarianas e HRR.^14 - 16^

Uma vez que o valor de HB é afetado por sangramento e nutrição, e o valor de RDW é afetado por inflamação e infecção, isso limita seu uso como fator prognóstico específico da doença sozinho. Portanto, estudos anteriores mostraram que o uso de HB e RDW juntos como HRR, em vez de usar qualquer um deles como fator prognóstico sozinho, aumenta a significância clínica.^11 - 16^ Embora a condição inflamatória básica, como sepse, tenha sido semelhante entre os grupos em nossos resultados, a menor HRR no hsPDA pode ser devida ao efeito do PCA na circulação, e não na inflamação. Assim, a HRR pode ser usada como um importante fator prognóstico que não é afetado por outros fatores de risco, incluindo a inflamação.^12^ De acordo com nossos resultados, foi revelado que a HRR era um parâmetro que também poderia ser usado para predizer hsPDA. No entanto, pensamos que a confirmação desses novos resultados com futuros estudos prospectivos seria benéfica.

O presente estudo é o primeiro a demonstrar a importância diagnóstica da HRR em prematuros com hsPDA. No entanto, nosso estudo atual tem algumas limitações. Uma dessas limitações é que a HRR é atualmente inválida para todos os prematuros devido ao desenho de estudo retrospectivo e de centro único. A segunda limitação é a ausência de um grupo controle de bebês nascidos a termo saudáveis. Além disso, estudos de eletroforese de hemoglobina que podem afetar os parâmetros eritrocitários não puderam ser realizados. Não realizamos parâmetros gasométricos. Finalmente, apenas os resultados do HRR na primeira amostra de sangue da veia umbilical pós-natal foram interpretados. Outros resultados de HRR em acompanhamentos diários não puderam ser avaliados.

## Conclusões

HB e RDW sozinhos não foram associados com hsPDA. No entanto, mostramos uma relação entre o valor de HRR no sangue do cordão umbilical e hsPDA. Usar HRR em vez de HB e RDW sozinho pode fornecer ao clínico informações valiosas para hsPDA. Também podemos supor que pode ser útil calcular a HRR em amostras de sangue coletadas durante o acompanhamento. Mais estudos são necessários para apoiar esta hipótese.
