# Supplementary material for: Proporção de Largura de Distribuição de Hemoglobina/Glóbulos Vermelhos (HRR): Um Novo e Promissor Parâmetro de Glóbulos Vermelhos no Fechamento Ductal
Source: Arq Bras Cardiol. 2023 Feb 13;120(2):e20220339. [Article in Portuguese] doi: 10.36660/abc.20220339 (PMC10263456; doi:10.36660/abc.20220339)
Supplement: Translation

# Translation: Hemoglobin/Red Cell Distribution width Ratio (HRR): A Novel and Promising Red Cell Parameter in Ductal Closure

**Authors:** Duran Yildiz, Ufuk Cakir, Ali Ulaş Tugcu, Burak Ceran, Cuneyt Tayman

**Affiliations:** 1 Turkey Ankara Bilkent City Hospital – Divisão de Neonatologia – Departamento de Pediatria – Universidade de Ciências da Saúde, Ankara – Turkey

**Keywords:** Heart Defecys, Congenital, Ductus Arteriosus, Patent, Hemoglobins, Infant, Premature

## Abstract

**Background:**

It is still unclear how effective hematological parameters are in the closure of patent ductus arteriosus (PDA).

**Objectives:**

The primary aim of our study is to investigate the effect of hemoglobin (HB)-to-red cell distribution width (RDW) ratio (HRR) on the closure of PDA.

**Methods:**

Premature babies with very low birth weight (VLBW: <1500 g) and <32 gestational weeks were included in the study, and all data were recorded retrospectively. Demographic characteristics, clinical results, red cell parameters, and HRR and their ratios were compared between hemodynamically significant PDA (hsPDA) and non-hsPDA groups. All results were statically analyzed, and P<0.05 was considered statistically significant.

**Results:**

A total of 677 premature babies, 269 in the hsPDA group and 408 in the non-hsPDA group, were included in the study. Hemoglobin (HB), hematocrit (HCT), mean cell volume (MCV), red blood cell (RBC), red cell distribution width (RDW), mean platelet volume (MPV), MCV/RBC ratio, HB/RBC ratio, RDW/RBC ratio, and RDW/MPV ratio were found to be similar between hsPDA and non-hsPDA groups, (p>0.05). HRR was found to be significantly lower in the hsPDA group [median (Quartile 1 (Q1) - Q3) (Q1 - Q3): 0.93 (0.8-1.0)] compared to non-hsPDA [median ( Q1 - Q3): 1.07 (1.0-1.2)] (p<0.001). The AUC for the diagnostic value of HRR in hsPDA was 0.816, and the cutoff value was ≤0.98 (p<0.001, 95% [CI]: 0.785-0.845, sensitivity: 90%, specificity: 92%).

**Conclusions:**

HRR value was found to be both an effective and powerful parameter in diagnosing hsPDA.

**Central Illustration:** : Hemoglobin/Red Cell Distribution width Ratio (HRR): A Novel and Promising Red Cell Parameter in Ductal Closure

Summary of the results of our study.

## Introduction

Patent ductus arteriosus (PDA) is premature infants’ most common cardiac disease. There is an increasing rate of morbidity and mortality related to PDA in premature infants. Left-to-right shunting due to PDA causes decreased systemic blood flow and increased pulmonary blood flow. As a result, there is an increased risk of serious morbidities such as pulmonary edema and bleeding, increased need for respiratory support, intraventricular hemorrhage (IVH), periventricular leukomalacia, necrotizing enterocolitis (NEC), retinopathy of prematurity (ROP), bronchopulmonary dysplasia (BPD), and poor neurodevelopmental outcomes.^1 , 2^

Failure of the closer of ductus arteriosus (DA) within 72 hours is defined as PDA. The frequency of PDA is inversely proportional to the gestational week (GW) and birth weight (BW). The incidence of PDA is 60-70% in premature infants under 28^th^GW and 20% in premature infants <32 weeks of gestation. PDA is seen at a rate of 40-55% in infants with a BW of <1000 g and 30% in babies with a BW of <1500 g.^3^

Functional closure of the DA occurs soon after birth due to increased oxygen saturation and decreased prostaglandin levels. Endothelial damage, subintimal disruption, platelet-endothelial interaction, neointimal proliferation, and vascular fibrous structure formation are effective in anatomical closure. Although the effects of GW, BW, and the above mechanisms on ductal closure are known, other mechanisms affecting ductal closure are not fully understood.^1^

Some markers and biochemical parameters may affect ductal closure.^4 - 6^Additionally, hematological parameters may affect ductal closure. Many studies evaluate the relationship between platelet count, indices and functions, and PDA.^1 , 7 , 8^However, few studies exist on the effect of erythrocytes and related parameters on ductal closure.^9 , 10^Currently, the effect of hematological parameters on PDA has not been fully elucidated. A small number of adult studies concluded that hemoglobin (HB)-to-red cell distribution width (RDW) ratio (HRR) might be a prognostic marker in some cancer types.^11 - 13^Recent studies have determined that low HRR value in adults with heart failure and coronary heart disease is an independent risk factor for mortality and adverse clinical outcomes.^14 - 16^However, whether HRR can be a predictor parameter in PDA has not been evaluated, which is the most common cardiovascular problem in newborns.

The clinical significance of HRR in PDA has not been previously evaluated. According to the hypothesis of our study, HRR derived from hematological parameters that can affect ductal closure may be a new promising parameter. The primary aim of this study is to evaluate the relationship between HRR and PDA. The secondary aim of our study is to evaluate the relationship between PDA and other red cell parameters in the complete blood count.

## Methods

### Study plan and patient selection

Premature infants with a BW of <1500 g and a GW of <32 weeks were eligible for the study. The study was retrospective, and data were obtained from the hospital’s medical records between September 2020 and November 2021. Patients with major congenital anomalies, congenital heart disease, perinatal asphyxia, birth weight ≥1500 g, and patients who died within the first three days after birth were excluded from the study. Demographic and clinical characteristics of the patients and red cell parameters in complete blood count were recorded. The patients were divided into hemodynamically significant PDA (hsPDA) and non-hsPDA. Ethical approval was obtained from the local hospital ethics committee before starting the study.

### Demographical and clinical characteristics

Gestational week, BW, gender, antenatal steroid administration, Apgar scores (at 1 and 5 minutes), small for gestational age (SGA), respiratory distress syndrome (RDS), IVH (≥3 grade), NEC (grade >2), moderate/severe BPD, ROP (requiring therapy), early-onset neonatal sepsis (EOS), late-onset neonatal sepsis (LOS), mechanical ventilation (MV), noninvasive ventilation (NIV) and oxygen duration, achieving full enteral feeding time (day), neonatal intensive care unit (NICU) stay duration, and mortality rates were recorded for all infants.

Patients requiring surfactant treatment were defined as RDS.^17^The diagnosis of severe IVH (≥3 grade) was demonstrated by cranial ultrasonography.^18^NEC classification (≥2 grade) was made based on clinical and laboratory findings.^19^BPD was defined as patients with a need for oxygen <30% (moderate) or receiving ≥30% oxygen or positive pressure (severe) at the 36^th^week of postmenstrual age.^20^Patients diagnosed with ROP according to the retinal examination performed by an ophthalmologist and subsequently treated were recorded.^21^Babies with a birth weight below the 10^th^percentile for GW were defined as SGA.^22^Postnatal sepsis was defined as EOS if it was ≤72 hours and LOS if it was >72 hours.^23^

### Diagnosis of hemodynamically significant patent ductus arteriosus

Following our unit protocol, all study patients receive Doppler echocardiography (ECHO) every 2 days during the first week, starting from the first 72 hours. The patients were diagnosed with hsPDA according to the clinical and ECHO criteria in Table 1 .^2^If the PDA internal diameter was <1.5 mm and/or the left atrium/aortic root ratio was <1.5 by ECHO, or if PDA was not detected, and if clinically and echocardiographically other than hsPDA, these patients were included in the non-hsPDA group. Patients in the hsPDA group have received medical therapy (nonsteroidal anti-inflammatory) at least once.^24^Those who did not respond to 2 courses of medical treatment were treated surgically. Fluid intake was initiated at 70-80 mL/kg per day in all infants and increased to a maximum of 150-160 mL/kg per day in increments of 10-20 mL/kg per day for all eligible patients.

### Complete blood count analysis

According to the unit protocol, blood samples from the umbilical vein were taken into ethylenediamine tetra-acetic acid (EDTA) tubes soon after delivery for a complete blood count. HB (g/dl), hematocrit (HCT, %), mean cell volume (MCV, fL), red blood cell (RBC, 10^6^/µL), RDW (%), and mean platelet volume (MPV) values were analyzed with Cell-Dyn 3700 automatic hemocytometer (Abbott, Abbott Park, IL, USA). The HRR value was obtained by dividing HB by RDW. The MCV/RBC ratio value was obtained by dividing MCV by RBC. The HB/RBC ratio was obtained by dividing HB by RBC. The RDW/RBC ratio was obtained by dividing RDW by RBC. The RDW/MPV ratio was obtained by dividing RDW by MPV.

### Statistical analysis

After the patient data were transferred to the computer environment, statistical analysis was performed with the Statistical Package for Social Sciences (SPSS), version 20.0 (SPSS Inc, Chicago, IL, USA) program. Visual (probability plots and histogram) and analytical methods (Kolmogorov-Smirnov Test) were used to evaluate the variables in terms of their conformity to the normal distribution. Fisher’s Exact test or Pearson Chi-Square test was used to evaluate categorical variables. An unpaired Student’s t-test or Mann-Whitney U test was applied for continuous variables. Normally distributed continuous variables were presented as mean ± standard deviation (SD), non-normally distributed variables were presented as median (Quartile 1 (Q1) - Quartile 3 (Q3)) (Q1 - Q3), and categorical variables were presented as frequency. Receiver operating characteristics (ROC) curves analysis was performed. After ROC analysis, the area under the curve (AUC) and the AUC’s 95% confidence interval (CI) were calculated. The threshold of HRR value for ductal closure was defined. Sensitivity and specificity were determined for the threshold of HRR value. If the obtained P value was below 0.05, it was considered statistically significant. The size of the sample was introduced for the primary endpoint as ductal closure; 124 patients in each group would have 80% power for detecting a 25% between-group difference (from 60 to 85%) in the percentage of permanent closures, using a two-sided, continuity-corrected χ^2^test at a significance level of 0.05.^8^

## Results

A total of 677 VLBW infants who met the inclusion criteria were allocated to the study group. 269 patients were included in the hsPDA group and 408 in the non-hsPDA group. In the hsPDA group, respiratory support (MV, NIV, and oxygen support), achieving a full enteral feeding day, and NICU stay were longer. RDS, BPD, ROP, IVH, and mortality frequency were significantly higher (p<0.05). There was no difference between the two groups regarding antenatal steroid administration (p>0.05). Other demographic and clinical characteristics were similar between the groups (p>0.05) ( Table 2 ). Among the hematological parameters, only HRR was found to be significantly lower in the hsPDA group than in the non-hsPDA group (p<0.001). The results of other hematological parameters were similar between the groups (p>0.05) ( Table 3 , Figure 1 ). The AUC value for the diagnostic value of HRR in hsPDA was 0.816, and the cutoff value was ≤0.98 (p<0.001, 95% [CI]: 0.785-0.845, sensitivity: 90%, specificity: 92%) ( Figure 2 ). Additionally, the results of our article were summarized in the central figure.

**Figure 1:** – Hematological ratios in groups with and without hemodynamically significant patent ductus arteriosus. HB: hemoglobin; hsPDA: hemodynamically significant patent ductus arteriosus; MCV: mean cell volume; MPV: mean platelet volume; RBC: red blood cell; RDW: red cell distribution width; *Statistically significant p values are highlighted.

**Figure 2:** – Receiver operating characteristic curves for HRR in hsPDA. hsPDA: hemodynamically significant patent ductus arteriosus; HRR: hemoglobin-to-red cell distribuition width ratio.

## Discussion

The primary aim of our study was to evaluate the relationship of HRR with hsPDA. This study found that the HRR value in the complete blood count was the most valuable parameter for determining hsPDA, with a high AUC, specificity, and sensitivity among the red cell parameters. If the HRR value was ≤9.8, it was found to be highly significant for the risk of hsPDA. However, no relationship was found between hsPDA and other red cell parameters, which was our secondary goal. Additionally, the duration of respiratory support, full enteral feeding day, and ICU stay were longer in infants with hsPDA. The most important risk factors for premature morbidity and mortality were GW and BW, as GW and BW were found to be similar in both groups. The increased rate of premature morbidity (RDS, BPD, ROP, IVH) and mortality in the hsPDA group seemed to be due to the effect of hsPDA. Therefore, our results supported the knowledge that hsPDA could increase premature morbidity and mortality in premature babies.^2 , 3^

If HB and HCT values decrease, tissue hypoxia increases. It is suggested that ductal closure will decrease with the deepening of hypoxia. However, the effect of HB and HCT values on hsPDA is not fully known.^2 , 3^Joye et al.^10^reported an increase in the frequency of hsPDA in the presence of severe anemia requiring postpartum transfusion. In the same study, it was interpreted that a higher and more stable HB level in the first ten days of life may positively affect the expected closure of the DA. In addition, in the mentioned study, HB level was found to be >13.5 g/dL in the non-hsPDA group. Therefore, the authors suggested that the closure of the DA may be supported by a higher HB level or maintenance of a certain HB level during the transition period. Our study found no relationship between HB and HCT values and hsPDA. This result can be explained according to Joye’s hypothesis, as the HB value was >13.5 g/dL in both groups of patients.^10^According to these results, the increased need for transfusion in patients with severe anemia increases the frequency of hsPDA and may decrease the response of hsPDA to the treatment.

The effect of MCV on ductal closure is unknown. We determined that MCV was not associated with hsPDA. Thus, it was concluded that erythrocyte volume did not affect ductal closure. In addition, we could not find any correlation between RBC value and hsPDA. The relationship between RBC and PDA has not been evaluated before. However, Bin-Nun et al.^25^found the absolute nucleated RBC count to be significantly higher in the hsPDA group than in the non-hsPDA group. It has been speculated that this result is due to high absolute nucleated RBC and ductal patency after delivery due to exposure to fetal hypoxia.^25^Another red cell parameter, RDW, reflects the heterogeneity in erythrocyte volume. In their study involving 41 premature babies, Garofoli et al.^26^reported that patients with hsPDA had a higher RDW value, and there was an inverse relationship between RDW values and GW.^26^The increased RDW in patients with hsPDA may be due to the higher frequency of LOS in the group with hsPDA. The increase in RDW is thought to occur due to inflammatory cytokines that inhibit erythrocyte maturation and accelerate the transition of younger and larger reticulocytes to peripheral circulation.^27^Previous studies, including higher numbers of patients, declared no relationship between RDW and hsPDA. In these studies, it was declared that the frequency of sepsis was similar between the groups as parallel to our results.^3 , 17^Therefore, RDW seems to be primarily affected by sepsis-related inflammation rather than hsPDA.^27^The increase in RDW is believed to be due to inflammatory cytokines that inhibit erythrocyte maturation and accelerate the transition of younger and larger reticulocytes to the peripheral circulation.27 Previous studies, including a larger number of patients, declared that there was no relationship between RDW and hsPDA.

The possible relationship between the MCV/RBC ratio, HB/RBC ratio, RDW/RBC ratio, and RDW/MPV ratio with any disease of the newborn has not been investigated. It has been found that MCV/RBC can be used effectively to differentiate iron deficiency anemia and thalassemia in childhood.^28^It has also been shown that RDW/RBC ratio can be used as an effective parameter for differentiating beta-thalassemia and iron deficiency.^29^The RDW/RBC ratio has also been reported to be used as an α-thalassemia screening test.^30^In addition, it has been stated that both RDW/RBC ratio and RDW/MPV can be used as important indicators in diagnosing complicated acute appendicitis in childhood.^31^The HB/RBC ratio has only been studied in liver toxicity. It has been shown that the severity of liver toxicity and the HB/RBC ratio value is directly proportional, and it is an effective parameter in demonstrating the hemostatic process.^32^

In our study, the relationship between MCV/RBC ratio, HB/RBC ratio, RDW/RBC ratio, and RDW/MPV ratio with hsPDA could not be demonstrated. The parameters used in all these ratios are calculated over the volume or distribution width of the erythrocyte. The volume or distribution width of the erythrocyte changes in diseases where inflammation is at the forefront, including sepsis. Therefore, these ratios are likely to be an indicator of inflammation. In our results, the sepsis incidence was similar in groups with and without hsPDA. This result suggested that the frequency of inflammation was similar between the groups. Our results supported the information that MCV/RBC ratio, HB/RBC ratio, RDW/RBC ratio, and RDW/MPV ratio were particularly affected in diseases caused by inflammation.^27^Therefore, the researches continue for other related and predictive hematological parameter that is not affected by inflammation for PDA, which is primarily affected by GW and BW and has a mixed pathophysiology. The fact that the required parameter is associated with tissue oxygenation, which has a significant effect on the closure of the DA, may increase the use value of this parameter. Therefore, the HB value has a parallel relationship with tissue oxygenation; it can be a safe parameter in evaluating the relationship between HB and DA. As the relationship between HB and PDA is not known exactly, the effect of the parameters, including HB, on the closure process of the duct should be evaluated. In adults, the prognostic significance of HRR in cancer patients has been the subject of limited studies. It has been reported to be associated with a poor prognosis of cancer below certain threshold values for certain cancer types. For example, HRR values of <0.989 in esophageal squamous cell cancer, <1.037 in head–neck cancer, <0.94 in muscle-invasive bladder cancer, <0.948 in non-small cell lung cancer, and <1.01 in lung adenocarcinoma are independent indicators for poor prognostic factors of patients.^11 - 13 , 33 , 34^In the study of Rahamim et al.,^14^in which 6888 adult patients with heart failure were evaluated, it was reported that HRR was a stronger parameter in mortality predictivity than HB or RDW alone.^14^Low HRR may be an independent and strong predictor of mortality and adverse clinical outcomes in coronary heart disease. In addition, HRR <9.76 was found to be the threshold value for the state of frailty in coronary heart diseases.^15 , 16^According to our results, the HRR value of <0.98 obtained from the umbilical cord was the most significant parameter to predict hsPDA. On the other hand, while HB or RDW alone was not significant in predicting hsPDA, it was found that the HRR value obtained from the ratio of these two parameters could be the more significant and strongest erythrocyte-derived marker.

In previous studies in cancer patients, it has been reported that HRR is an indicator of immune, nutritional and inflammatory status.^11^When HB and RDW were used as prognostic factors in studies on cancer types, no significant difference was found in overall survival. Instead of evaluating HB and RDW alone, as in our results, their clinical value becomes meaningful when it is used as HRR, which is a ratio to each other.^34^Similar results were obtained in studies evaluating the relationship between heart failure and coronary heart diseases and HRR.^14 - 16^

Since the HB value is affected by bleeding and nutrition, and the RDW value is affected by inflammation and infection, it limits its use as a disease-specific prognostic factor alone. Therefore, previous studies have shown that using HB and RDW together as HRR rather than using either as a prognostic factor alone increases the clinical significance.^11 - 16^While the basic inflammatory condition, such as sepsis, was similar between the groups in our results, the lower HRR in hsPDA may be due to the effect of PDA on circulation rather than inflammation. Thus, HRR can be used as an important prognostic factor that is not affected by other risk factors, including inflammation.^12^According to our results, it was revealed that HRR was a parameter that could also be used to predict hsPDA. However, we thought confirming these new results with future prospective studies would be beneficial.

The present study is the first to demonstrate the diagnostic importance of HRR in premature infants with hsPDA. However, our current study has some limitations. One of these limitations is that the HRR is currently invalid for all premature infants due to the single-center and retrospective study design. The second limitation is the absence of a healthy-term infant control group. In addition, hemoglobin electrophoresis studies that may affect erythrocyte parameters could not be performed. We did not perform blood gas parameters. Finally, only the results of the HRR in the first postnatal umbilical vein blood sample were interpreted. Other HRR results at daily follow-ups could not be evaluated.

## Conclusions

HB and RDW alone were not associated with hsPDA. However, we showed a relationship between the HRR value in cord blood and hsPDA. Using HRR instead of HB and RDW alone can provide the clinician with valuable information for hsPDA. We can also assume that it may be useful to calculate the HRR in blood samples taken during follow-up. Further studies are needed to support this hypothesis.

**Table 1:** – Hemodynamically significant patent ductus arteriosus

**Clinical features**	Murmur
	Hyperdynamic precordium
	Bounding preductal pulses
	Worsening respiratory status
	Wide pulse pressure
	Hypotension
	Metabolic acidosis
**Echocardiographic features**	Increased left atrium to aortic root ratio
	Cardiomegaly
	Left-to-right shunting
	Large open ductus (>1.5 mm)
	Reversal of flow in postductal major arteries

**Table 2:** – Demographical and clinical characteristics of hsPDA and non-hs PDA groups. *Statistically significant p values are highlighted

Variables	hsPDA (n: 269, 39.7%)	Non-hsPDA (n: 408, 60.3%)	p
Gestational age, weeks^a^	28.2± 2.0	28.5 ± 2.1	0.089
Birth weight, g^a^	1041 ± 220	1080 ± 248	0.103
Male gender^b^	129 (47.9)	220 (53.9)	0.388
Antenatal steroid^b^	183 (69)	290 (71)	0.929
Apgar score at 1 min^c^	5 (4-6)	5 (5-6)	0.112
Apgar score at 5 min^c^	7 (6-8)	7 (7-8)	0.208
Oxygen duration, days^c^	35 (12-48)	12 (5-29)	<0.001*
NIV duration, days^c^	10 (4-17)	4 (1-6)	<0.001*
MV duration, days^c^	2 (0-17)	0 (0-2)	<0.001*
ENS^b^	6 (2.2)	9 (2.2)	0.671
LOS^b^	75 (27.8)	81 (19.8)	0.086
RDS^b^	217 (80.6)	199 (48.7)	<0.001*
BPD^b^	79 (29.3)	36 (8.8)	<0.001*
ROP^b^	36 (13.3)	19 (4.6)	<0.001*
IVH (grade≥3)^b^	37 (13.7)	19 (4.6)	<0.001*
NEC (grade≥2)^b^	7 (2.6)	7 (1.7)	0.114
Full enteral feeding, day^c^	15 (6-21)	13 (11-17)	0.001*
ICU stay, days^c^	67 (45-80)	51 (36-66)	0.001*
Mortality^b^	45 (16.7)	53 (12.9)	0.007*

*^*a*^mean ± standard deviation,^*b*^n (%),^*c*^median (Quartile 1 (Q1) - Quartile 3 (Q3)) (Q1 - Q3). BPD: bronchopulmonary dysplasia; ENS: early neonatal sepsis; IVH: intraventricular hemorrhage; LOS: late-onset sepsis; MV: mechanical ventilation; NEC: necrotizing enterocolitis; NICU: neonatal intensive care unit; NIV: noninvasive ventilation; hsPDA: hemodynamically significant patent ductus arteriosus; RDS: respiratory distress syndrome; ROP: retinopathy of prematurity.*

**Table 3:** – Hematological parameters of hsPDA and non-hs PDA groups

Variables	hsPDA (n: 269, 39.7%)	Não-hsPDA (n: 408, 60.3%)	p
HB, (g/dL)^a^	15.7 (14.4-16.9)	16.7 (15.5-18.1)	0.352
HCT, (%)^a^	48 (44.2-51.0)	51.4 (47.9-55.2)	0.736
MCV, (fL)^a^	114 (109-119)	115 (110-120)	0.146
RBC, (106/µL)^a^	4.17 (3.7-4.4)	4.4 (4.1-4.8)	0.711
MPV, (fL)^a^	7.5 (7.1-8.3)	7.6 (7.4-8.4)	0.321
RDW, (%)^a^	15.9 (15.2-16.5)	15.9 (15.2-16.6)	0.367
HB/RDW ratio,^a^	0.93 (0.8-1.0)	1.07 (1.0-1.2)	<0.001*
MCV/RBC ratio,^a^	27.9 (24.9-30.7)	25.6 (23.0-28.6)	0.061
HB/RBC ratio,^a^	3.7 (3.5-3.9)	3.7 (3.5-3.9)	0.666
RDW/RBC ratio,^a^	2.62 (3.5-4.2)	2.60 (3.3-3.9)	0.102
RDW/MPV ratio,^a^	1.69 (1.8-2.2)	1.68 (1.8-2.1)	0.213

*Statistically significant p values are highlighted.^*a*^median (Quartile 1 (Q1) - Quartile 3 (Q3)) (Q1 - Q3). HB: hemoglobin; HCT: hematocrit; hsPDA: hemodynamically significant patent ductus arteriosus; MCV: mean cell volume; MPV: mean platelet volume; RBC: red blood cell; RDW: red cell distribution width.
